# Metformin for the management of tuberous sclerosis: What does the evidence tell us?

**DOI:** 10.17179/excli2021-4213

**Published:** 2021-09-27

**Authors:** Anirudh Srinivasan, Beti Naga Shashi Kiran, Abdulla Koduvath, Auroprakash Pal, Ananthu Suresh, Amudhan Kannan, Mirunalini Ravichandran

**Affiliations:** 1 Jawaharlal Institute of Postgraduate Medical Education and Research (JIPMER), Puducherry, India

## ⁯⁯

***Dear Editor,***

Tuberous sclerosis (TS) is a genetic disorder that affects multiple systems in the body, which includes: the central nervous system, skin, renal system, and heart. TS has an overall incidence of 1 in 6,000 live births (Osborne et al., 2000[5]). It is an autosomal dominant disorder involving the TSC1 and TSC2 gene mutations with variable expressivity. These mutations lead to the inhibition of proteins (hamartin and tuberin) that play a crucial role in the inhibition of mTOR protein kinase, which is involved in the development of tumors. Studies in the literature have reported that these mutations, by inhibiting the proteins mentioned above, cause disinhibition of mTOR protein kinase, thereby affecting the multiplication and growth of the cells (Curatolo and Moavero, 2012[4]). The most important and the most common system that is involved in TS is the central nervous system. Patients with TS often present with seizures as the most common clinical manifestation (Thiele, 2004[6]; Connolly et al., 2006[3]). Hamartomas in the brain result in refractory seizures, which are difficult to manage with anti-epileptic drugs and thus requiring surgical management. Subependymal giant cell astrocytoma (SEGA) is a serious manifestation of TS that can lead to life-threatening complications and sudden death. SEGA contributes to high morbidity and mortality in patients with TS. Patients with seizures face adverse effects due to anti-epileptic therapy, which further adds to morbidity and mortality. Therefore, it is crucial to diagnose and manage patients with TS at earlier stages to reduce the seizure relapses and avoid life-threatening complications due to SEGA.

One drug that has demonstrated an inhibitory effect on mTOR protein kinase is metformin (Amin et al., 2019[1]). Metformin, the first-line drug used to treat type 2 diabetes mellitus, has shown various properties that can be utilized to manage various disorders. Metformin has also shown anti-cancer properties in multiple studies, and ongoing studies are being done to determine its potential usage in the management of cancers. One of the randomized controlled trials (RCT) that studied the effect of metformin in patients with tuberous sclerosis is the RCT by Amin et al. (2021[2]). We congratulate the authors of Amin et al. RCT for the great efforts put in for this study. This was a double-blinded RCT which was done to determine if metformin would reduce the growth of hamartomas in patients with TS. A total of 55 patients with TS aged over ten years were randomly assigned either to the metformin group (n = 28) or the placebo group (n = 27).

Of these, four patients were not included in the analysis due to withdrawal from the RCT. The two groups received metformin or placebo for one year. The change in volume of renal angiomyolipomas (AML), SEGA, and frequency of seizures were studied at the end of one year of therapy. Out of the patients who received metformin, 14 patients had SEGA. In the group that received placebo, 13 patients had SEGA. Metformin showed a significant reduction in the volume of SEGA. In the metformin group, 12 had a history of seizures, while 9 had a history of seizures in the placebo group. The Metformin group showed a significant reduction (43.7 %) in seizures compared to the 3.1 % reduction in the placebo group. The authors concluded that metformin has the potential to reduce the SEGA volume in younger patients. It was also concluded that metformin appeared to reduce seizures in patients with TS. This study also reported three serious adverse events in the metformin group (Amin et al., 2021[2]).

This double-blinded RCT is very important for the literature because not many studies or RCTs studied the effect of metformin in the management of TS. Metformin, being a widely available drug, can be considered for the management of TS. The addition of metformin will also help manage underlying asymptomatic or symptomatic conditions such as type 2 diabetes mellitus and polycystic ovarian syndrome. Future RCTs to determine the efficacy and safety of metformin in patients with TS with large sample size and long-term follow-up are necessary for literature.

## Conflict of interest

The authors declare no conflict of interest.
